# *Aspergillus calidoustus*: An Emerging Cause of Invasive Aspergillosis and the Role of Metagenomic Next-Generation Sequencing Test in Its Diagnosis

**DOI:** 10.1155/crdi/3221057

**Published:** 2025-11-29

**Authors:** Tulika Chatterjee, Moni Roy, Mohammad O. Almoujahed, Sharjeel Ahmad

**Affiliations:** Department of Internal Medicine, University of Illinois College of Medicine, Peoria, Illinois, USA

**Keywords:** *Aspergillus calidoustus*, invasive aspergillosis, invasive aspergillosis in immunocompromised, Karius test, metagenomic next-generation sequencing test for fungal infection, neuro-aspergillosis

## Abstract

Transplant recipients have a high risk of infection with opportunistic pathogens. The type, dose, and duration of immunosuppression and use of prior broad-spectrum antimicrobials contribute to overall risk of infections. Aspergillosis is a known opportunistic infection that can occur as mid or late infection after visceral transplant. *Aspergillus fumigatus* is the commonly isolated species, but with the use of prophylactic broad-spectrum antifungals, other species such as *Aspergillus calidoustus* are emerging. We report a case of invasive sinusitis and brain mass due to this species of Aspergillus that was identified using next-generation sequencing (NGS). Use of NGS early in clinical presentation may help in effective management of opportunistic infections in immunocompromised hosts.

## 1. Case Presentation

A 19-year-old male was admitted with fever, nausea, vomiting, diarrhea, mild abdominal pain, poor oral intake, and acute kidney injury. He was diagnosed with hepatocellular carcinoma at the age of 17 years followed by multivisceral (liver, pancreas, small bowel, and colon) transplant. Since then, the patient had recurrent episodes of rejection, skin, and colonic graft versus host disease, chronic kidney disease, and post-transplant lymphoproliferative disorder (PTLD). For PTLD, he was treated with cyclophosphamide, rituximab, and prednisone followed by chimeric antigen receptor (CAR) T-cell therapy with cyclophosphamide, doxorubicin hydrochloride, vincristine sulfate, and prednisone (CHOP regimen). He also developed obstructive lung disease. Five months prior to this admission, the patient was diagnosed with rhino-orbital sinusitis and underwent septoplasty. Fungal culture of nasal tissue grew *Rhizopus oryza*e and blood next-generation sequencing (NGS) of microbial cell-free DNA test (Karius test, Karius Inc, Redwood City, CA) at that time was positive for *Aspergillus fumigatus* and *Rhizopus oryzae*. (1 ⟶ 3)-β-D-Glucan levels (Fungitell) were elevated at  > 500 pg/mL (normal  < 60 pg/mL). He received intravenous (IV) L-amphotericin for a week followed by isavuconazole which was later transitioned to oral posaconazole. Fungitell levels did drop to 241 pg/mL after 2 months of antifungal treatment. He was continued on home oral posaconazole on this admission. One week prior to the current admission, he was briefly hospitalized and treated properly for Influenza A with hypogammaglobulinemia. He received oseltamivir for Influenza A and IV immune globulin infusion.

At the time of this admission, he was febrile with a temperature of 103 degrees Fahrenheit and was normotensive. He was now 28 months status post multivisceral transplant. He was on tacrolimus and prednisone for transplant immunosuppression. He was on azithromycin, minocycline, trimethoprim/sulfamethoxazole (TMP/SMX), and valacyclovir for prophylaxis. He was also on posaconazole for chronic fungal sinusitis at the time of this admission. He was found to have hypomagnesemia, hypophosphatemia, hypokalemia, and bilateral multifocal pneumonia. Piperacillin-tazobactam IV was started empirically. Fluids and electrolyte replacement was initiated. Further testing showed presence of cytomegalovirus (CMV) viremia with CMV DNA quantitative polymerase chain reaction (PCR) 7,859,836 IU/mL (normal value < 1 IU/mL). Biopsies taken during EGD and colonoscopy showed presence of CMV infection in samples taken from stomach and colon. He was started on IV Ganciclovir. Aspergillus antigen index level was elevated at  > 3.750 (normal  < 0.5 index).

His respiratory status deteriorated, and he developed acute respiratory distress syndrome (ARDS) requiring intubation. Blood cultures and bronchoalveolar lavage (BAL) fluid culture grew TMP/SMX-resistant *Stenotrophomonas maltophilia*. Repeat testing showed high-level CMV viremia. Colonoscopy and EGD were repeated which showed rare CMV viral inclusion bodies. Ophthalmology evaluation did not show the presence of CMV retinitis. Moxifloxacin and minocycline for *Stenotrophomonas maltophilia* bacteremia and pneumonia were initiated. Fungitell levels were 225 pg/mL on admission with repeat testing upon clinical deterioration showing an increased level of  > 500 pg/mL. He was initially started on micafungin to provide broader antifungal coverage.

**Figure 1:**
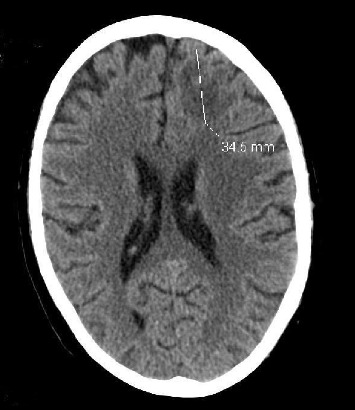
Axial CT head without contrast showing low attenuation involving predominantly the white matter of the left frontal lobe shown with measurements. No shift of the midline structures was noted.

**Figure 2:**
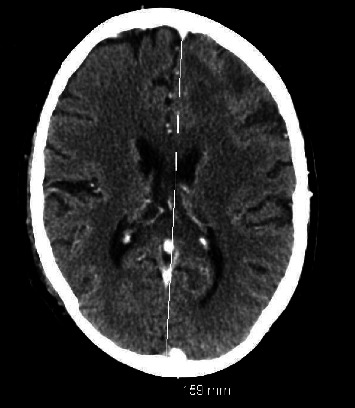
Follow-up axial CT head without contrast after 3 days showed left frontal horn mass effect and 3–4 mm of left-to-right shift of the third ventricle. White line showing midline shift.

The patient remained critically ill with medical issues including respiratory failure with prolonged intubation and renal failure requiring dialysis. Due to altered mentation, with a history of invasive fungal infection, a computed tomography (CT) head was done that showed sinus opacification and frontal lobe mass. Repeat head CT scans showed progression of mass with areas of worsening infraction (Figure 1 and Figure 2).

Serum Aspergillus antigen was persistently positive at  > 3.750 index. With worsening clinical status and uptrending Fungitell, micafungin was soon discontinued due to poor CNS penetration and posaconazole was switched to isavuconazole and the patient was started on liposomal Amphotericin. Karius test was sent again and returned positive for *Aspergillus calidoustus*. Repeat Fungitell level was 423 pg/mL. Given the progression of mass lesion as noted on CT, azole-resistant *Aspergillus calidoustus* infection was suspected and antifungal regimen was switched to IV micafungin, voriconazole, and terbinafine based on limited data available for treatment of such infection.

The Karius test reports results in DNA molecules per microliter (MPM). The patient had an *Aspergillus calidoustus* load of 10,244 MPM (reference range  < 10 MPM). Other pathogens were detected with stenotrophomonas maltophilia (97,188 MPM, reference  < 84 MPM), CMV (14,490 MPM, reference  < 10 MPM), BK polyoma virus (1,533 MPM, reference  < 10 MPM). With patients' known history of stenotrophomonas infection, CMV viremia, and BK viremia, the NGS testing positive for the other pathogens was as expected.

**Figure 3:**
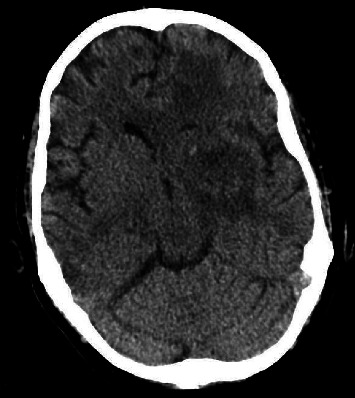
Final CT axial head without contrast showed increased mass effect with decreased attenuation within the left frontal lobe as compared to prior imaging results.

Despite aggressive antifungal regimen for 3 days, his medical condition continued to deteriorate and the patient became unresponsive. Repeat CT head (Figure 3) showed a new area of hemorrhage near the left caudate head with increasing mass effect.

**Figure 4:**
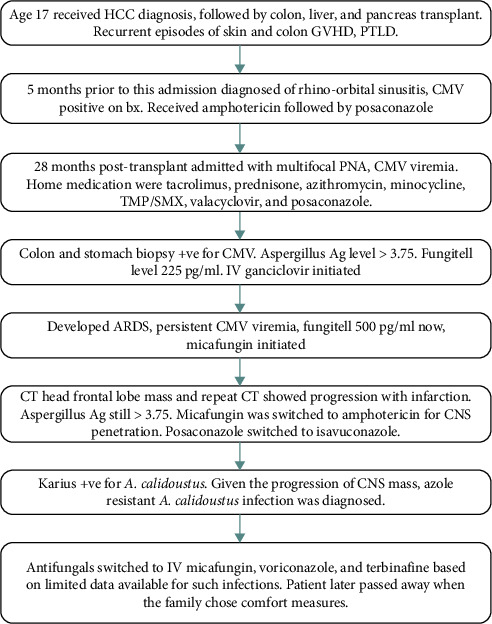
Flow diagram showing timeline of events. Abbreviations: PTLD: post-transplant lymphoproliferative disorder; HCC: hepatocellular carcinoma; GVHD: graft versus host disease; bx: biopsy; pna: pneumonia; CMV: cytomegalovirus; ARDS: acute respiratory distress syndrome; CNS: central nervous syndrome; TMP/SMX: trimethoprim-sulfamethoxazole.

A family meeting was held regarding goals of care discussion, and a decision was made to proceed with comfort-focused care. The patient passed away the next day. The flow diagram is shown in the timeline of events (Figure 4).

## 2. Discussion

Invasive aspergillosis (IA) is characterized by tissue invasion, particularly vascular invasion by Aspergillus species (a group of filamentous fungal mold found in soil and decomposing matter, endemic worldwide), which can lead to contiguous or hematogenous dissemination and multi-organ involvement. It is the most common mold infection in patients of solid organ transplant (SOT), hematopoietic stem cell transplant (HSCT) and acute leukemia on chemotherapy [1]. The EORTC/MSGERC (European Organization for Research and Treatment of Cancer/Mycoses Study Group of the National Institute of Allergy and Infectious Diseases initially classified invasive fungal diseases into possible, probable and proven. The definition was modified in 2003, expanding the diagnosis of proven invasive fungal disease including individuals regardless of immune status [2]. Based on the revised definition, the patient met the criteria for proven invasive fungal infection with amplification of fungal DNA by PCR combined with DNA sequencing when molds are seen in formalin-fixed paraffin-embedded tissue [3].

IA is a significant cause of morbidity and mortality in solid organ transplant and stem cell transplant recipients especially in the first year of a transplant, likely due to a more intense regimen of immunosuppression. Approximately 9.3% to 16.9% of deaths in the first year of transplant have been associated with IA. Historically, *A. fumigatus, Aspergillus flavus, A. Niger* are the most prevalent causes of IA [4]. The most common route of infection is airborne exposure to Aspergillus spores and spore germination in the sino-pulmonary tract. Pneumonia, tracheobronchitis and invasive sinusitis are usual presentations.

Due to the invasive nature of the organism a prompt initiation of antifungal therapy is needed. Our patient was diagnosed with fungal sinusitis prior to this presentation and was on posaconazole prior to this admission. Triazoles, especially voriconazole or posaconazole are used for antifungal prophylaxis in the transplant or chemotherapy patients [1].

*Aspergillus calidoustus* is a newly identified species of the *Aspergillus* genus causing IA. It causes IA in immunocompromised patients, mainly seen in patients with HSCT and to a lesser extent in patients of solid organ transplant [4]. *A. calidoustus* can cause invasive infection of the central nervous system [5]. It is found to be resistant to azoles and has been reported to be the cause of breakthrough infections in immunocompromised patients who are on prophylactic antifungal therapy [6]. Egli et al. noted that the incidence of *A. calidoustus* infection occurred at a higher number in lung transplant patients on voriconazole prophylaxis, as they noted that infection due to A. *calidoustus* increased from 0/1000 patients-years to 11.3/1000 since 2008 and suspected selection pressure leading to emergence of azole-resistant strains. Also, in this study, a decrease in cases with *Aspergillus fumigatus* from 73.9/1,000 to 49/1,000 was noted [4]. In their case-control study, they reported eight cases of IA, and six of them had prior azole exposure for varying periods of time either for primary post-transplant fungal prophylaxis or for secondary prophylaxis for a previous Aspergillus infection, which was the same for our patient [4]. *A. calidoustus* exhibits intrinsic reduced susceptibility to azoles and that partly explains the emergence of this species. Our patient was treated for *A. fumigatus* associated with invasive rhino-orbital fungal infection. He received liposomal amphotericin B followed by isavuconazole and was on posaconazole at the time of his last admission which was later switched to isavuconazole.

*Aspergillus calidoustus* is phenotypically very similar to *Aspergillus utus* with the difference that *A. calidoustus* can grow at 37 degrees Celsius [4]. It is possible that it may have been misreported as *Aspergillus ustus* in the past [1, 6], so the exact incidence of *A. calidoustus* is not very clear [4]. Similar to our patient, *Tyll* et al. reported a fatal case of cerebral aspergillosis with *A. calidoustus* in an immunocompromised male [6].

With the advancement in transplant technologies, streamlining of organ donation, more patients are able to receive organ transplantation and are subsequently placed on immunosuppressive medications and prophylactic antimicrobials. This can create selection pressure and cause the emergence of more resistant opportunistic organisms. It is thus critical to diagnose the Aspergillus species accurately and perform in vitro susceptibilities in cases of IA in these immunocompromised patients.

The NGS test used for pathogen detection in our patient was Karius Spectrum (Karius, Inc, Redwood City, California, USA). It is a commercially available metagenomic NGS test that can identify cell-free microbial DNA (cfDNA) from over 1,200 pathogens, including fungi, bacteria, parasites, and DNA viruses. The test uses Illumina platform.

The test can detect genomic material from pathogens in plasma which could be helpful in the diagnosis of invasive fungal infections [7, 8]. This test has a rapid turn-around time, a potential to avoid invasive diagnostic biopsy, and early pathogen species identification. It could also lead to a decrease in unnecessary exposure to empiric broad-spectrum antibiotic treatment for immunocompromised patients while waiting for traditional culture results. NGS of cfDNA has another advantage of detecting coinfections in immunocompromised patients [8]. With the emergence of newer cryptic species of Aspergillus, the traditional identification markers of colony morphology and microscopic characteristics are not completely reliable. Identification via molecular markers is a more consistent method of speciation [9]. The limitations of use of this test are the cost and unavailability of the test worldwide. Karius Spectrum can also check for antimicrobial resistance for 18 bacterial pathogens and 4 classes of antimicrobial resistance: methicillin-resistant Staphylococci (SCCmec, mecA, and mecC), vancomycin-resistant Enterococci (vanA and vanB), carbapenem-resistant Gram-negative bacteria (KPC), extended spectrum beta-lactamase (ESBL)–producing Gram-negative bacteria (CTX-M). It is however unable to test for antifungal resistance in fungal pathogens. Another major limitation of using this test is that it may detect cfDNA from stress reactivation of certain organisms which are not producing disease in certain patients, and thus the results need to be interpreted by an expert, based on clinical context.

Negri et al. noted in their study that *A. calidoustus* had high MIC values against three azoles: voriconazole, posaconazole, and itraconazole (4, 4, and 32 g/mL, respectively) [9]. MIC of *A calidoustus* to these azoles has been typically reported to be over 4 mg/dL [10]. Gampedakis et al. reported an in vitro synergistic effect of voriconazole and terbinafine with therapeutic range concentrations as compared to monotherapy or other combinations of antifungals [5]. The combination synergistic effect (fractional inhibitory concentration index <=0.5) in 9 out of 10 *A. calidoustus* strains was however studied on nonvertebrate larvae, and the human doses were extrapolated [5]. Mendoza et al. reported a case of successful treatment of *A. calidoustus* IA treated successfully with a combination regimen of isavuconazole and terbinafine [10]. Panackal et al. in their study of 15 systemic cases of *Aspergillus ustus* reported that several patients who received the combination regimen of voriconazole and caspofungin had *A. ustus* infection resolved, but it was not clear if this was related to drug synergy [11]. In our young patient who was critically ill with invasive fungal infection leading to progressing brain mass, an antifungal regimen of micafungin, voriconazole, and terbinafine was tried, but unfortunately, he died shortly after this antifungal regimen was started.

It is apparent that the mortality is high in cases of IA due to *A. calidoustus* in these complicated, severely immunocompromised patients with multiple comorbidities who are at very high risk for other opportunistic infections as well. An earlier diagnosis in our case could have led to a more favorable outcome. We suggest the early use of NGS for diagnosis of opportunistic infections in immunocompromised patients.
